# Face (e)motion and the third visual pathway

**DOI:** 10.1073/pnas.2513881122

**Published:** 2025-07-28

**Authors:** Aina Puce

**Affiliations:** ^a^Department of Psychological and Brain Sciences, College of Arts and Sciences, Indiana University, Bloomington, IN 47405-7007

The paper by Yan et al. (see this issue pages XXX-YYY) entitled “*The Brain Computes Dynamic Facial Movements for Emotion Categorization Using a Third Pathway*” is a notable contribution to understanding visual processing of natural stimuli (1). Activation in a third visual pathway for social perception, proposed in 2021 by David Pitcher and the late Leslie Ungerleider (2) was studied. This pathway adds to the “what/where” visual pathways, proposed in 1982 by Ungerleider and Mishkin (3), to explain dynamic social interactions in human and nonhuman primates. We still do not fully understand how face-sensitive regions interact while viewing dynamic facial expressions in everyday social interactions. Yan et al. performed multiple behavioral and neurophysiological experiments with novel dynamic face stimuli. Creating realistic dynamic facial stimuli is difficult as the human face has over 40 muscles for displaying specific emotions (e.g., sadness) or emotion blends (e.g., unpleasant surprise) with varying intensity. The current study breaks ground with ecologically valid dynamic facial stimuli made with a generative model, using these methods to create customized stimuli for individual subjects.

Overall, this study is a methodological tour de force. The authors synthesized dynamic facial expression stimuli with a generative model based on different degrees of contraction (from 1 to 6) from 42 random individual Facial Action Units (AUs) over six face motion attributes (4), simulating actions of individual facial muscles making up the Facial Action Coding System (5). The overall stimulus set of 2,400 animations was used in an initial behavioral study where 10 subjects categorized each animation, selecting one of six basic emotions, using a seventh option of “don’t know” if unsure what expression was presented. An established model-fitting procedure (4) derived an individualized emotion recognition model for each subject of the main AUs associated with the six basic emotions, creating a 600-item stimulus set for a subsequent magnetoencephalography (MEG) experiment for the same 10 subjects. In this high-resolution neurophysiological study, subjects viewed individualized dynamic emotion stimuli and identified six emotions (similar to the behavioral task, with a seventh response category for “other”). Neural sources of MEG signals were identified as a function of time. Source activity was averaged over selected anatomical parcels in the three visual pathways and occipital cortex. Emotion identity was classified from MEG activity using a Mutual Information (MI) procedure (6), expressing temporal MI activation profiles in cortical parcels in the occipital cortex and the three visual pathways (7).

Yan et al. lead the way for sophisticated behavioral and high temporal and spatial resolution neurophysiological testing of individual subjects.

**Fig. 1.**
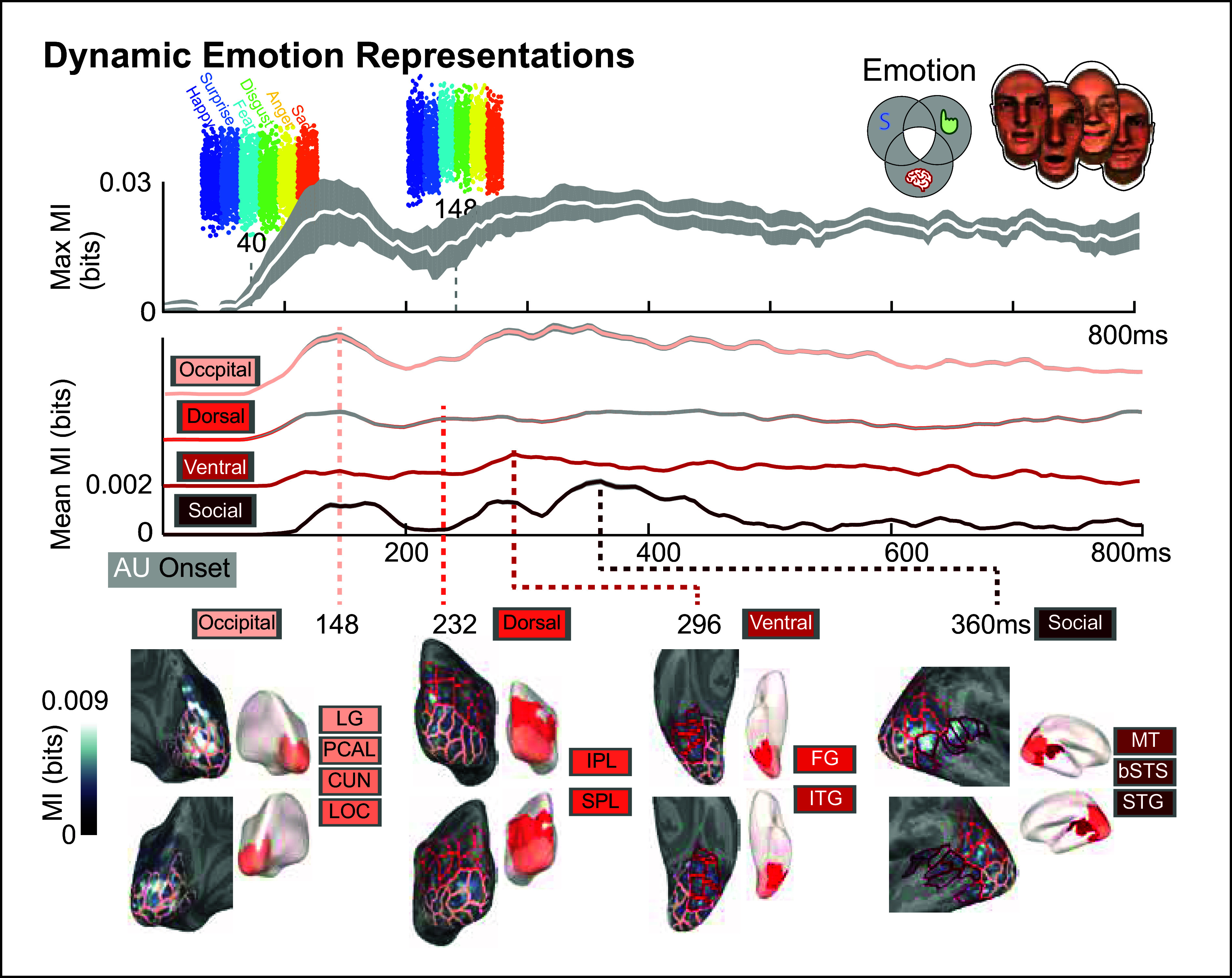
Dynamic emotion representations. (*Top*) Maximum MI (in bits) averaged across six emotions and 10 subjects (white curve) and all neural sources. Single trial MEG responses for emotions at two time points are color-coded. Early in time (i.e., 40 ms), emotions are not differentiated, whereas they are at 148 ms (emotions with largest responses are fear and disgust). (*Middle*) Curves show average MI values for 102 subregions in visual pathways (social, ventral, dorsal) and occipital cortex. For high MI values, MEG responses distinguish between emotions. When low, there are no MEG differences between emotions. (*Lower*) For occipital cortex and three pathways, gray inflated brains (*Left*) show MI peak times at 148 (occipital), 232 (dorsal), 296 (ventral), and 384 ms (social) with color-coded annotation for selected subregions that match those in the *Middle* panel. The reference white/red-colored brains (*Right*) indicate progression of modeled emotion representations across different brain regions. LEGEND: lingual gyrus (LG), pericalcarine (PCAL), cuneus (CUN), lateral occipital cortex (LOC), inferior parietal lobe (IPL), superior parietal lobe (SPL), fusiform gyrus (FG), inferior temporal gyrus (ITG), medial temporal gyrus (MTG, including MT/V5), bank of superior temporal sulcus (bSTS), superior temporal gyrus (STG). Image credit: reprinted from ref. 1.

MI analysis of MEG data by rated emotion category showed distinctive temporal profiles in the visual pathways (Fig. 1). The *Top* panel shows maximum MI (white trace) averaged across 10 subjects and all active neural sources, showing how neural activity can be differentiated emotions at different time points. The *Middle* panel breaks down mean MI plotted by color-coded sources in each visual pathway (and occipital region). The *Lower* panel depicts selected brain parcels (and overall) regions in each color-coded visual pathway and occipital region displayed as inflated mediolateral, posterior, ventral, and lateral views of posterior brain. Peak time points (relative to animation onset) of mean MI indicate progressive dynamics in each visual pathway at 148 ms (occipital), 232 ms (dorsal), 296 ms (ventral), and 384 ms (third or social).

The pattern of propagation of activity from the occipital cortex was notable. Propagation to the third pathway reached motion-selective middle temporal visual area (MT/V5) and subsequently the facial motion-selective superior temporal sulcus (STS) and gyrus (8). Propagation to the motion-sensitive dorsal pathway spread to inferior and superior parietal lobules. Importantly, activation did not spread (as expressed by MI) to inferior temporal gyrus—to a pathway processing unchangeable aspects of the face.

This latter finding is interesting, given there are no known direct white matter paths between human STS (third pathway) and FG (ventral pathway) (9, 10). Indeed, earlier face processing models have posited inferior occipital gyrus (IOG, not included here) as the node between ventral and dorsal pathways (11, 12). That said, human intracranial field potentials and white matter tract endpoint data suggest that the inferior temporal cortex may mediate between STS and FG (13). Both IOG and inferior temporal cortex react more strongly to dynamic versus static stimuli, and STS reacts earlier to dynamic stimuli. Hence, activation of occipital regions and third pathway structures, such as STS, to dynamic facial stimuli (13) is consistent with the MI profiles reported by Yan et al.

Historically, opinion has been divided on where STS sits in the visual pathway scheme: Is it in the dorsal pathway—with MT/V5, or is it an intermediary between dorsal and ventral pathways (14)? The third (social) pathway proposal evolved partly to solve this conundrum (2). Importantly, MI data here are consistent with the three visual pathway scheme—timing of MI profiles separates activity in MT/V5 and STS (third pathway) from that of inferior and parietal lobules (dorsal pathway) and from structures of the ventral pathway.

In their review, Pitcher and Ungerleider (2021) emphasized right hemisphere biases in the third visual pathway to social signals, in line with earlier mainly right-sided findings in STS, superior temporal gyrus, and MT/V5 (2). Yan et al. suggest that right hemispheric areas may resolve uncertainty when ambiguous AU signals are encountered. Their reasoning is based on MI profiles and confusion matrices for the six emotion ratings. If their speculation is accurate, then better emotion categorizations, especially for emotion blends could occur, where ambiguity may be maximal.

Context provides another source of ambiguity for interpreting emotional facial expressions. In real life, directed and averted gaze provides social context, i.e., to whom the expression is being directed at and who will likely be the target of a subsequent action (2, 8). In the current study, avatar’s gaze was directed at the observer. Specifically, gaze aversion produces larger brain responses when social meaning is not being explicitly evaluated (14). An interesting outstanding question is whether ambiguity of some emotional expressions might be reduced if seen in avatars with an averted gaze? In the current study, one could imagine viewers observing a face “reacting” to an “unseen” individual. While scanning visual scenes in everyday life, additional context also comes from parafoveal (15) and extrafoveal vision. Perhaps these multiple visual context questions might be lines of investigation for future studies?

An additional visual pathway idea per se is not novel (see ref. 14). Category-selectivity for dynamic visual (and auditory) stimuli including living things (animals) and manipulable objects such as tools (16) is known. These active brain areas lie within the lateral occipitotemporal cortex, leading to the proposal for a more general third visual pathway (14), devoted to reading dynamic reactions from human faces, hands, and bodies (2), as well as animals, and dealing with mobile inanimate objects, such as tools. Injury to these brain regions impairs recognition of these stimuli, while leaving basic low-level motion processing intact (17, 18). A posterior–anterior complexity gradient in the human temporal lobe exists for processing naturalistic dynamic human stimuli, e.g., for more communicative actions (19). Brain regions for human voice processing also sit in superior temporal regions (20). Indeed, future research on the “third visual pathway” should extend to other sensory modalities.

Yan et al. lead the way for sophisticated behavioral and high temporal and spatial resolution neurophysiological testing of individual subjects. In our network science era, future studies could test how active networks in the three visual pathways sequence interactions via specific white matter pathways (21) during multimodal dyadic encounters.

This is an exciting time to study how our brains integrate activity within category-selective visual and auditory brain regions during naturalistic interactions. Writing this commentary has been bittersweet: Leslie Ungerleider is no longer with us to discuss these exciting new developments in social and cognitive neuroscience.
